# Non-invasive monitoring of pH and oxygen using miniaturized electrochemical sensors in an animal model of acute hypoxia

**DOI:** 10.1186/s12967-021-02715-7

**Published:** 2021-02-04

**Authors:** Laura Pla, Sergio Berdún, Mònica Mir, Lourders Rivas, Sandrine Miserere, Samuel Dulay, Josep Samitier, Elisenda Eixarch, Miriam Illa, Eduard Gratacós

**Affiliations:** 1 BCNatal | Fetal Medicine Research Center (Hospital Clínic and Hospital Sant Joan de Déu), Universitat de Barcelona, Barcelona, Spain; 2 Centro de Investigación Biomédica en Red en Bioingeniería, Biomateriales Y Nanomedicina (CIBER-BBN), Monforte de Lemos 3-5, Pabellón 11, 28029 Madrid, Spain; 3 Nanobioengineering Group, Institute for Bioengineering of Catalonia (IBEC) Barcelona Institute of Science and Technology (BIST), 12 Baldiri Reixac 15-21, 08028 Barcelona, Spain; 4 Department of Electronics and Biomedical Engineering, University of Barcelona, Martí I Franquès 1, 08028 Barcelona, Spain; 5 Institut D’Investigacions Biomèdiques August Pi I Sunyer (IDIBAPS), Barcelona, Spain; 6 Centre for Biomedical Research On Rare Diseases (CIBER-ER), Barcelona, Spain; 7 Institut de Recerca Sant Joan de Déu, Esplugues de Llobregat, Spain

**Keywords:** Acute hypoxia-acidosis, Continuous monitoring of acid–base status, High-risk pregnancies, Electrochemical sensors

## Abstract

**Background:**

One of the most prevalent causes of fetal hypoxia leading to stillbirth is placental insufficiency. Hemodynamic changes evaluated with Doppler ultrasound have been used as a surrogate marker of fetal hypoxia. However, Doppler evaluation cannot be performed continuously. As a first step, the present work aimed to evaluate the performance of miniaturized electrochemical sensors in the continuous monitoring of oxygen and pH changes in a model of acute hypoxia-acidosis.

**Methods:**

pH and oxygen electrochemical sensors were evaluated in a ventilatory hypoxia rabbit model. The ventilator hypoxia protocol included 3 differential phases: basal (100% FiO_2_), the hypoxia-acidosis period (10% FiO_2_) and recovery (100% FiO_2_). Sensors were tested in blood tissue (ex vivo sensing) and in muscular tissue (in vivo sensing). pH electrochemical and oxygen sensors were evaluated on the day of insertion (short-term evaluation) and pH electrochemical sensors were also tested after 5 days of insertion (long-term evaluation). pH and oxygen sensing were registered throughout the ventilatory hypoxia protocol (basal, hypoxia-acidosis, and recovery) and were compared with blood gas metabolites results from carotid artery catheterization (obtained with the EPOC blood analyzer). Finally, histological assessment was performed on the sensor insertion site. One-way ANOVA was used for the analysis of the evolution of acid-based metabolites and electrochemical sensor signaling results; a t-test was used for pre- and post-calibration analyses; and chi-square analyses for categorical variables.

**Results:**

At the short-term evaluation, both the pH and oxygen electrochemical sensors distinguished the basal and hypoxia-acidosis periods in both the in vivo and ex vivo sensing. However, only the ex vivo sensing detected the recovery period. In the long-term evaluation, the pH electrochemical sensor signal seemed to lose sensibility. Finally, histological assessment revealed no signs of alteration on the day of evaluation (short-term), whereas in the long-term evaluation a sub-acute inflammatory reaction adjacent to the implantation site was detected.

**Conclusions:**

Miniaturized electrochemical sensors represent a new generation of tools for the continuous monitoring of hypoxia-acidosis, which is especially indicated in high-risk pregnancies. Further studies including more tissue-compatible material would be required in order to improve long-term electrochemical sensing.

## Background

Hypoxia during the prenatal period has been found to be related to perinatal morbidity and mortality [1]. Several conditions have been associated with fetal hypoxia, with placental insufficiency being one of its most prevalent causes. Placental insufficiency causes an inadequate supply of oxygen to the fetus and, depending on the degree of insufficiency, the severity of fetal hypoxia and its consequences may differ. Whereas acute and severe episodes of hypoxia have been related to intrauterine death, periods of moderate hypoxia that persist over time may also have deleterious consequences for fetal growth and development [2]. Both acute and chronic prenatal hypoxia induce fetal anabolic metabolism followed by fetal hypoxemia and acidosis [3, 4]. Fetal hemodynamics tries to compensate for this situation by redistributing the blood flow to vital organs such as the cerebrum and myocardium [5, 6]. Doppler ultrasound evaluation could identify these hemodynamical changes [7] and has also been described as a useful tool for fetal monitoring as it has been correlated with blood gas results [8].

As there is as yet no effective intrauterine treatment able to overcome fetal hypoxia-acidosis, Doppler monitoring would allow us to plan elective termination of the pregnancy when the risk of intrauterine death outweighs the risk of prematurity [9]. However, one of the major limitations of Doppler ultrasound evaluation is that it cannot be performed continuously. The development of a medical device able to detect the fetal hypoxia-acidosis status in a precise and continuous manner would be a big step forward in the clinical managing of this population. To this end, previous evidence has demonstrated the feasibility of sensor devices being inserted in different tissues, including in the vascular system [10–13], the brain [14–17], the gastric system [18], subcutaneously and intramuscularly [19–22] to monitor and detect acid–base status. However, most of these sensors are not designed to be used during the intrauterine period. Rivas et al. have recently developed a miniaturized electrochemical sensor to be inserted into the fetal intramuscular compartment that has demonstrated a good accuracy in detecting hypoxia and acidosis in in vitro, ex vivo, and in vivo models [23].

In this study, as a first step we tested the hypothesis that previously developed miniaturized electrochemical sensors [23] could detect and monitor oxygen and pH changes continuously in a model of acute hypoxia-acidosis. We evaluated and compared the performance of an electrochemical sensor inserted intramuscularly (in vivo sensing) or immersed in blood (ex vivo sensing). Finally, in a subgroup of animals, the pH electrochemical sensor signal was recorded 5 days after the insertion (day 5). Histological analysis of the tissue around the electrochemical sensors was collected and examined in order to evaluate tissue biocompatibility. This study was intended as a preceding step to the evaluation of the performance of the electrochemical sensors in fetal tissue.

## Methods

### Animals: ethics, housing, and preparation

Animal handling and all experimental procedures were performed in accordance with applicable regulation and guidelines and with the approval of the Animal Experimental Ethics Committee of the Universitat de Barcelona (ref. 236/16) and the competent authority Generalitat de Catalunya (ref. 9349).

A total of 27 New Zealand White male rabbits (2.5–3 kg body weight) were included in this study; 19 out of 27 animals were included in the short-term electrochemical sensor evaluation (day 0) and the remaining 8 were used for the long-term (day 5) evaluation. The animals were acclimated for 5 days before surgery and were individually caged (dimensions 70 × 75 × 40 cm) with visual, auditory, and olfactory contact allowed between different individuals. Housing was provided under conventional conditions in an environmentally controlled room (temperature: 16.9–23 °C; humidity: 35–85%; photoperiod: 12:12 h light:dark cycle). Animals had free access to tap water, a standard commercial pelleted diet (2030 Teklad global rabbit diet, Envigo), and hay.

### Miniaturized electrochemical sensors: pH and pO_2_

Electrochemical sensors were designed as previously described [23]. Briefly, the oxygen electrochemical sensor consisted of 3 electrodes of 15 cm in length. One electrode was a bare platinum wire used as a counter, another was a modified silver wire (Ag/AgCl) used as a pseudo-reference electrode (PRE), and the last was a membrane-modified platinum wire with Nafion used as a working electrode (WE). Two layers of Nafion perfluorinated resin solution 5 wt. % were deposited on the platinum wire and dried overnight in an argon chamber. The wire was then cured at 100 °C for 1 h. Nafion offers biocompatibility and provides a protective layer to the sensor that reduces the diffusion of small neutral or negatively charged interfering species such as uric acid and ascorbic acid [24]. All 3 electrodes were assembled within a 5–7 mm piece of polyether ether ketone (PEEK) tubing and the final diameter was ~ 500 µm. Oxygen electrochemical sensors were pre-calibrated in PBS buffer using different oxygen concentrations (pre-calibration analyses). The electrochemical measurements were performed using a multipotentiostat from PalmSens (Germany) and the sensor signal was detected by electric current (amperometry, nA). O_2_ dissolved in liquid or gas sample is an electroactive molecule and the sensor uses this characteristic for its detection. The O_2_ cathodic reduction produces a current that is proportional to the O_2_ concentration. The reduction mechanism of oxygen may follow either the two- or four-electron pathway. The latter is attributed to materials such as platinum, used in the sensors, in which O_2_ is reduced completely to water without intermediate steps [25]. In our system, the reduction of O_2_ was induced by applying − 0.7 V and the current produced was measured by chronoamperometry.

A total of 18 oxygen electrochemical sensors were used for this study: 6 of them were evaluated in the ex vivo setting and the other 12 were evaluated in vivo in the short-term period.

The pH electrochemical sensor consisted of 2 electrodes of 15 cm in length, a PRE, and a WE. The PRE was a modified silver wire (Ag/AgCl) and the WE was a membrane-modified platinum wire with a polypyrrole film obtained by electropolymerization. This process was performed by cyclic voltammetry on the platinum electrode from 0.45 to 0.95 V for 3 continuous scans at a scan rate of 0.025 V·s^−^ in a solution of 0.1 M of pyrrole and 0.1 M KCl as a supporting electrolyte in MilliQ filtered water. Before use, the pH sensor was immersed in a 0.1 M HCl solution for 48 h for pre-conditioning. The polypyrrole deposited on the pH sensor surface undergoes protonation/deprotonation depending on the surrounding pH. The fluctuation of the proton charges at the WE are detected by differential potential with respect to the PRE. Both electrodes were also assembled within a 5–7 mm piece of PEEK tubing achieving a final diameter of ~ 500 µm and pre-conditioned in a 0.1 M HCl solution for 48 h prior to use. pH electrochemical sensors were pre-calibrated with standard commercial solutions from 4.006 to 7.413 (pre-calibration analyses). As with the oxygen sensors, the electrochemical measurements were performed using a multipotentiostat, however, in this case the sensor signal was detected by electric potential (voltage, mV). A total of 29 pH electrochemical sensors were used for this study: 3 of them were evaluated in the ex vivo setting, 10 were evaluated in vivo in the short-term period, and the other 9 sensors were evaluated in vivo in the long-term period.

### Short-term evaluation

#### General anesthesia

Ketamine and xylazine (35 and 5 mg/kg, respectively, SC) were first administrated and followed by general anesthesia with isofluorane (2%) in oxygen (100%, 2 L/min) using a face mask. A peripheral ear venous catheter was placed and body core temperature was maintained with an electric pad. Heart and respiratory rate, oxygen saturation, body core temperature, and reflexes were monitored and recorded during the whole experiment.

#### Electrochemical sensor implantation

Sensor implantation for in vivo evaluation was carried out as follows: A skin incision around 3 cm long at the level of the right femoral quadriceps muscle was performed. After exposing the muscle, 1–2 electrochemical sensors per rabbit were implanted through a 2–3 mm muscle incision and secured to the muscle using simple knots (silk, 3/0). The skin was closed using a running suture (silk, 3/0). The 15 cm long electrodes attached to each electrochemical sensor were allowed to be externalized through the skin incision and connected to a portable electrochemical device for continuous monitoring.

Sensor implantation for ex vivo evaluation was carried out as follows: During the ventilatory hypoxia induction protocol (see below), an additional 0.2 mL blood sample in each interval was collected for the ex vivo analyses. For that purpose, electrochemical sensors (pO_2_ and pH) were immersed in the blood collected at the different time points and the sensor signals were recorded.

#### Ventilatory hypoxia induction protocol and monitoring

Ventilatory hypoxia was started immediately after the muscle electrochemical sensor insertion and under the general anesthesia explained above. The neck region was shaved and a midline incision in the ventral cervical area was performed. The trachea was then carefully dissected and exposed. A 3–4 mm tracheostomy was performed and a 3.0 endotracheal tube was inserted. Ventilatory hypoxia-acidosis was induced using a mechanical ventilator (SAR-1000 ventilator and CTP-VA-3 valve assembly from CWE, Inc.). The mechanical ventilation parameters were changed depending on the phase of the experiment (basal registration, hypoxia-acidosis induction, recovery). Rocuronium bromide (0.6 mg/kg, IV) was administered when needed. The carotid artery was also carefully exposed through the same ventral cervical incision and cannulated with a 22G-IV for serial blood sampling.

During the basal registration (the first 15 min), respiratory ventilatory parameters were set at 20 breaths per minute, a 1:2 inspiration/expiration (I/E) ratio and a 2 L/min inhalatory flux with 100% oxygen. During this period, 2 blood samples (0.2 mL) were obtained at 15-min intervals from the carotid artery for the evaluation of acid-based metabolites (pO_2_, pH, bicarbonate, potassium, and lactate) using EPOC blood analysis (EPOC reader and EPOC BEGM test card, Alere/Siemens Healthcare, Barcelona). Changes in the amperometric or potentiometric signals obtained from the oxygen and pH sensors, respectively, inserted in the muscular tissue or immersed in the blood collected were continuously recorded and compared with the acid-based metabolite results (in vivo sensing and ex vivo sensing).

After 15 min of basal registration, acute hypoxia-acidosis was induced for 75 min by reducing the respiratory rate, the liters per minute and the fraction of inspired oxygen (FiO_2_). Respiratory ventilatory parameters in this phase were set at 10 breaths per minute, a 1:2 I/E ratio and a 1 L/min inhalatory flux with 10% oxygen and 90% nitrogen. Similarly to the previous period, blood sampling was performed every 10–15 min for the evaluation of acid-based metabolites. The acid-based metabolite results were then compared with the readings of the intramuscular or blood sensors (in vivo sensing or ex vivo sensing).

Finally, the recovery period was induced, lasting for 30 min. The ventilatory parameters of the animals were the same as those used in the basal period. Blood sampling and sensor readings were also performed as previously explained.

### Long-term evaluation

#### Anesthesia, sensor implantation and ventilatory hypoxia protocol

General anesthesia and electrochemical sensor implantation for in vivo evaluation was performed as in the short-term evaluation protocol. However, in these animals, the 15 cm cables attached to each electrochemical sensor were inserted in the subcutaneous tissue (between muscle and skin) to prevent the animal from chewing them. The skin was then closed using an intradermal running suture (Vicryl, 3/0) and antibiotic prophylaxis (penicillin G, 300,000 IU) was administrated. General anesthesia was stopped and the animals were kept under a warming blanket until they awoke. Animal welfare and the integrity of the surgical wound were evaluated during the post-operative period. The animals received buprenorphine (0.04 mg/kg, SC, every 12 h) and meloxicam (0.3 mg/kg, SC, every 24 h) as a post-operative analgesia for 3 days. The ventilatory hypoxia induction protocol was performed 5 days after the insertion of the sensors (day 5) as described for the short-term evaluation.

#### Post-calibration of the electrochemical sensors in the long-term evaluation

In order to further evaluate the sensors’ viability after 5 days of tissue insertion, their in vitro functionality was re-tested after finalizing the ventilatory hypoxia protocol (post-calibration analyses). For this purpose, 2 of the total pH electrochemical sensors, selected at random, were removed and immersed in standard commercial solutions with a pH between 4.006 and 7.413. The signal of each electrochemical sensor was recorded and compared with those signals obtained before tissue insertion (pre-calibration analyses).

### Sampling and histological analyses

After finalizing the ventilatory hypoxia induction protocol, the animals were sacrificed with pentobarbital (200 mg/kg, IV) and the muscular tissue surrounding the insertion area of the sensors was carefully excided, fixed for one week by immersion in 10% buffered formalin and embedded in paraffin for further histological analyses. Histological analyses were performed in 7 samples obtained on the day of insertion (short-term evaluation) and 8 samples were obtained 5 days after insertion (long-term evaluation).

Paraffin blocks were serially cut in 5 μm thick transverse sections with a microtome and standard hematoxylin/eosin (Mayer’s Hematoyxlin, ref. 51275Sigma; Eosin, ref. 1.15935.0100, Merk) and masson trichrome staining (ref. HT15-1KT, Sigma) were performed. One representative section from each sample was examined under an optic microscope (Leica microsystem CH-9435, type: DFC425C). Tissue integrity and cell infiltration were analyzed with hematoxylin eosin staining, whereas masson trichome staining was used for the evaluation of fibrotic reaction.

**Fig. 1:**
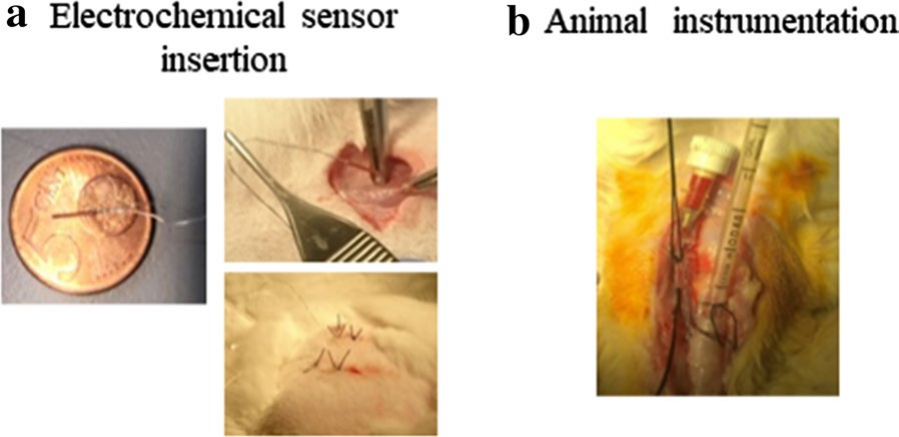
**a** Illustrative images of the electrochemical sensor insertion. **b** Illustrative images of the tracheostomy and the carotid artery catheterization

Electrochemical sensor implantation and animal instrumentation are represented in Fig. 1.

### Statistical analysis

Data were expressed as mean and standard error of the mean (SEM). A one-way ANOVA analysis with a Dunnett’s post-test was used for the analysis of the results of the evolution of acid-based metabolites and the electrochemical sensor signaling parameters during the ventilatory hypoxia model. A t-test was used for the pre- and post-calibration analyses and chi-square analyses were used for the categorical variables. A P value < 0.05 was considered statistically significant. All statistical analysis was performed using GraphPad prism 6.0 software.

## Results

### Acid-based metabolites results

Six of the 27 animals included in the study were discarded (4 animals from the short-term evaluation and 2 animals from the long-term evaluation) leaving a final sample size of 21. Reasons for the exclusions included failure to induce hypoxia-acidosis during the ventilatory hypoxia protocol (*n* = 4), pre-existing acidosis during the basal period (*n* = 1) and pre-surgical death (*n* = 1).

**Fig. 2:**
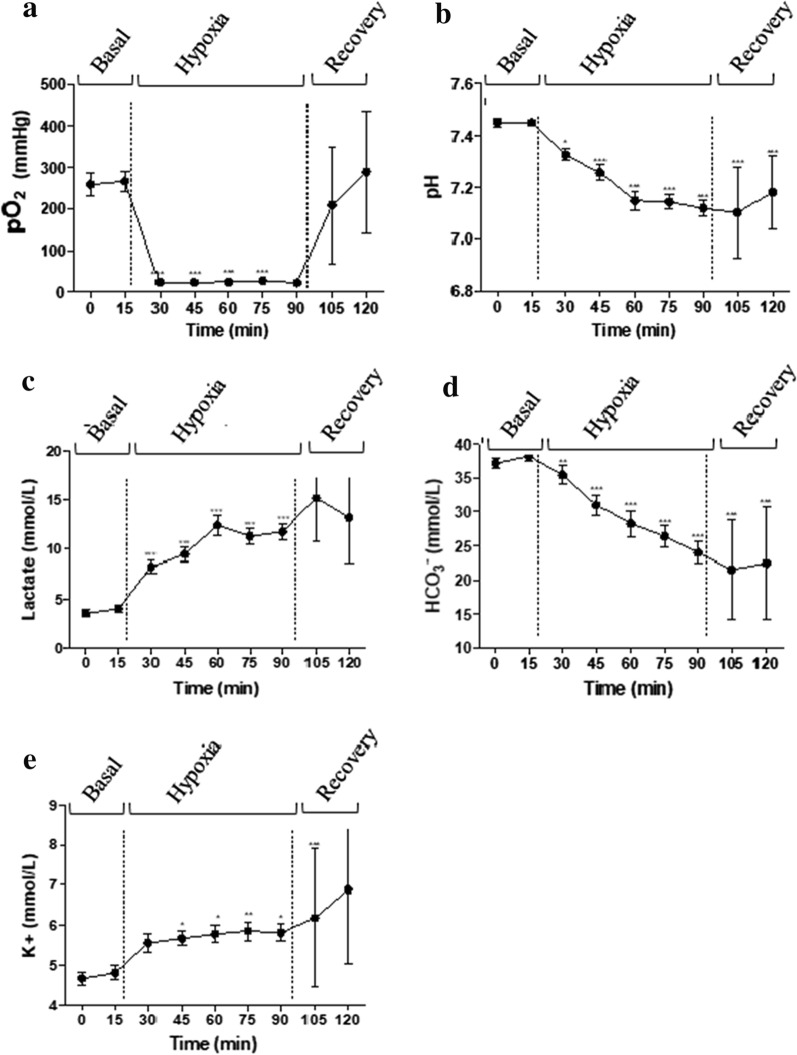
Evolution of arterial acid-based metabolites during the hypoxia induction protocol (basal, hypoxia-acidosis and recovery periods): Partial pressure of oxygen (pO_2_) (**a**), pH (**b**), lactate (**c**), bicarbonate (HCO3^−^) (**d**) and potassium (K +) (**e**) concentration. Data are expressed as mean ± SEM. Statistical significance was declared when *P < 0.05, **P < 0.01, ***P < 0.001 and ****P < 0.0001 between basal and each time. *N* = 21 animals

A description of the metabolite acid–base status results obtained by EPOC during the different phases of the study is detailed in Fig. 2. Briefly, during the basal period, metabolites were within the normal range of normoxia. As expected, during the hypoxia-acidosis period, a significant decrease in pO_2_, pH, and bicarbonate and a significant increase in lactate and potassium concentration in comparison with the basal period were observed. The pO_2_ decrease was the quickest, reaching its lowest value after 2.38 min, and it presented more pronounced changes in comparison with the rest of the metabolites that presented more progressive and less marked changes. Finally, in the recovery phase, pO_2_ was the only metabolite that reached similar levels to those reported in the basal period.

### Electrochemical sensors in the short-term evaluation

#### Oxygen electrochemical sensors: ex vivo and in vivo sensing

A total of 6 animals were included. For the ex vivo evaluation, 1 oxygen electrochemical sensor per animal was tested and in all of them an electrochemical sensor signal was obtained (100% functional success rate). For the in vivo evaluation, 2 oxygen electrochemical sensors per animal were implanted intramuscularly and functionally tested. Six of these 12 sensors did not detect any signal during the whole experiment, therefore the functional success rate was 50%.

**Fig. 3:**
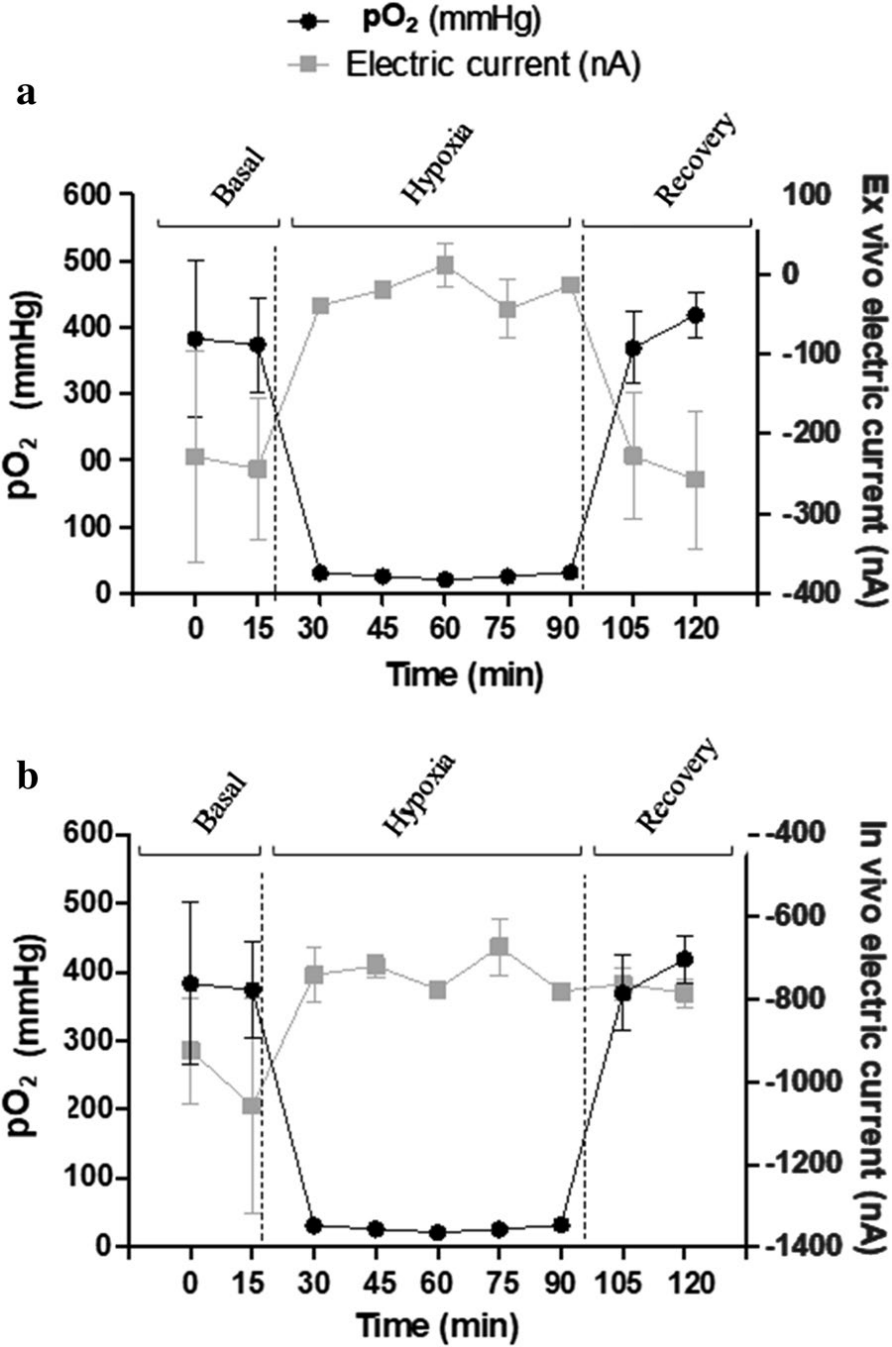
Evolution of partial pressure of oxygen (pO_2_) (measured by arterial gasometry) and electric current (nA) (measured by oxygen electrochemical sensors) in ex vivo (**a**) and in vivo (**b**) models during the ventilatory hypoxia induction protocol. Data are expressed as mean ± SEM. *N* = 6 animals, 6 electrochemical sensors tested ex vivo and 6 electrochemical sensors tested in vivo

Figure 3 depicts signal changes detected by the ex vivo and in vivo sensors during the different phases of the study. Overall, the basal signal of the electrochemical sensors differed between ex vivo and in vivo. The electric current in the ex vivo oxygen sensing was between 100 and − 400 nA, however, this signal was lower in the in vivo sensing (− 400 to − 1400 nA). In the hypoxia period, the electrochemical sensors detected the marked decline of the pO_2_ levels, although the intensity of the current signal differed depending on the tissue. Between 15 and 90 min, there was a significant decrease of pO_2_ (91.4% decrease) in both evaluations (in vivo and ex vivo). Coinciding with this pO_2_ decrease, both sensors presented a significant increase in the electrochemical signal, although this increase was higher in the ex vivo sensing in comparison with the in vivo (94.38 vs 26.3%, p < 0.01). In the recovery period, only the electric signal from the ex vivo evaluation returned to values similar to those detected during the basal period.

#### *pH electrochemical sensors: *ex vivo* and *in vivo* sensing*

A total of 9 animals were included. We tested 3 pH electrochemical sensors in 3 different animals with a 100% functional success rate. For the in vivo evaluation, 1–2 electrochemical sensors per animal were implanted intramuscularly and functionally tested. Three electrochemical sensors did not detect any signal during the whole experiment. Therefore, the rate of functional success was 70% for in vivo pH sensing.

**Fig. 4:**
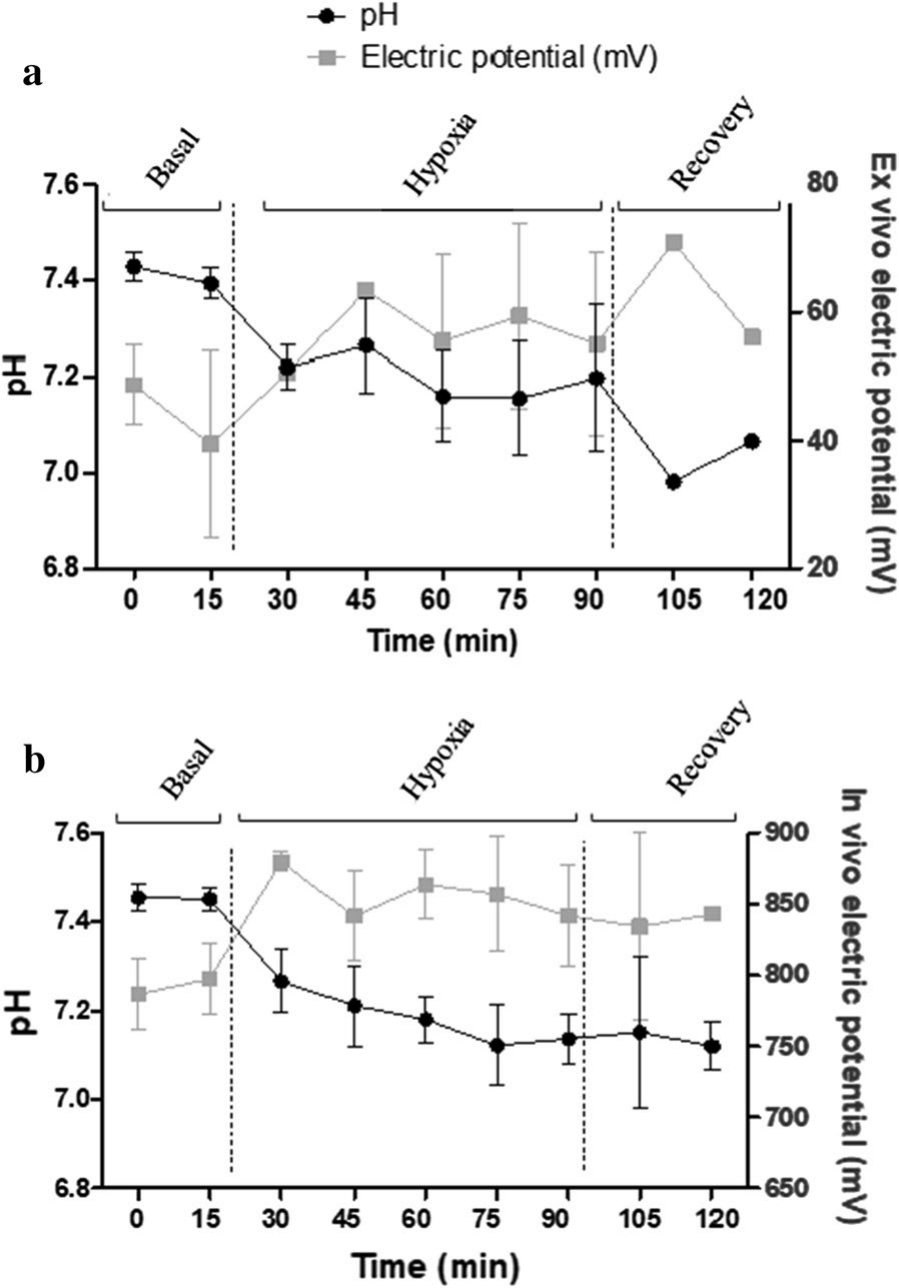
Evolution of pH (measured by arterial gasometry) and electric potential (mV) (measured by pH electrochemical sensors) in ex vivo (**a**) and in vivo (**b**) models during the ventilatory hypoxia induction protocol. Data are expressed as mean ± SEM. *N* = 9 animals, 3 electrochemical sensors tested ex vivo and 7 electrochemical sensors tested in vivo

A description of the signal detected by the ex vivo and in vivo sensing during the different phases of the study is detailed in Fig. 4. Briefly, the basal signal of the electrochemical sensors differed between ex vivo and in vivo. The basal electric potential in the ex vivo pH sensing was between 80 and 20 mV; however, the signal was higher in the in vivo evaluation (900–650 mV). In the hypoxia period both signals (ex vivo and in vivo) detected the progressive decline in pH, although the intensity of the current’s electrochemical signal differed depending on the tissue. Between the time points of 15 and 90 min, coinciding with the pH decrease, both sensors detected an increase in the electrochemical signal, although this increase was higher in the ex vivo sensor in comparison with the in vivo (39.3 vs 4.2%, P < 0.01). In the recovery period, only the signal from the ex vivo evaluation seemed to detect the pH changes.

#### pH electrochemical sensors in the long-term evaluation

A total of 6 animals were included for pH sensing in the long-term period. The 9 pH electrochemical sensors (1–2 electrochemical sensors per animal) were implanted intramuscularly and functionally tested at day 5. One electrochemical sensor was excluded owing to technical problems with the computer. Of the remaining 8 implanted sensors, 1 did not detect any signal during the whole experiment. Therefore, the rate of functional success was 87.5%.

**Fig. 5:**
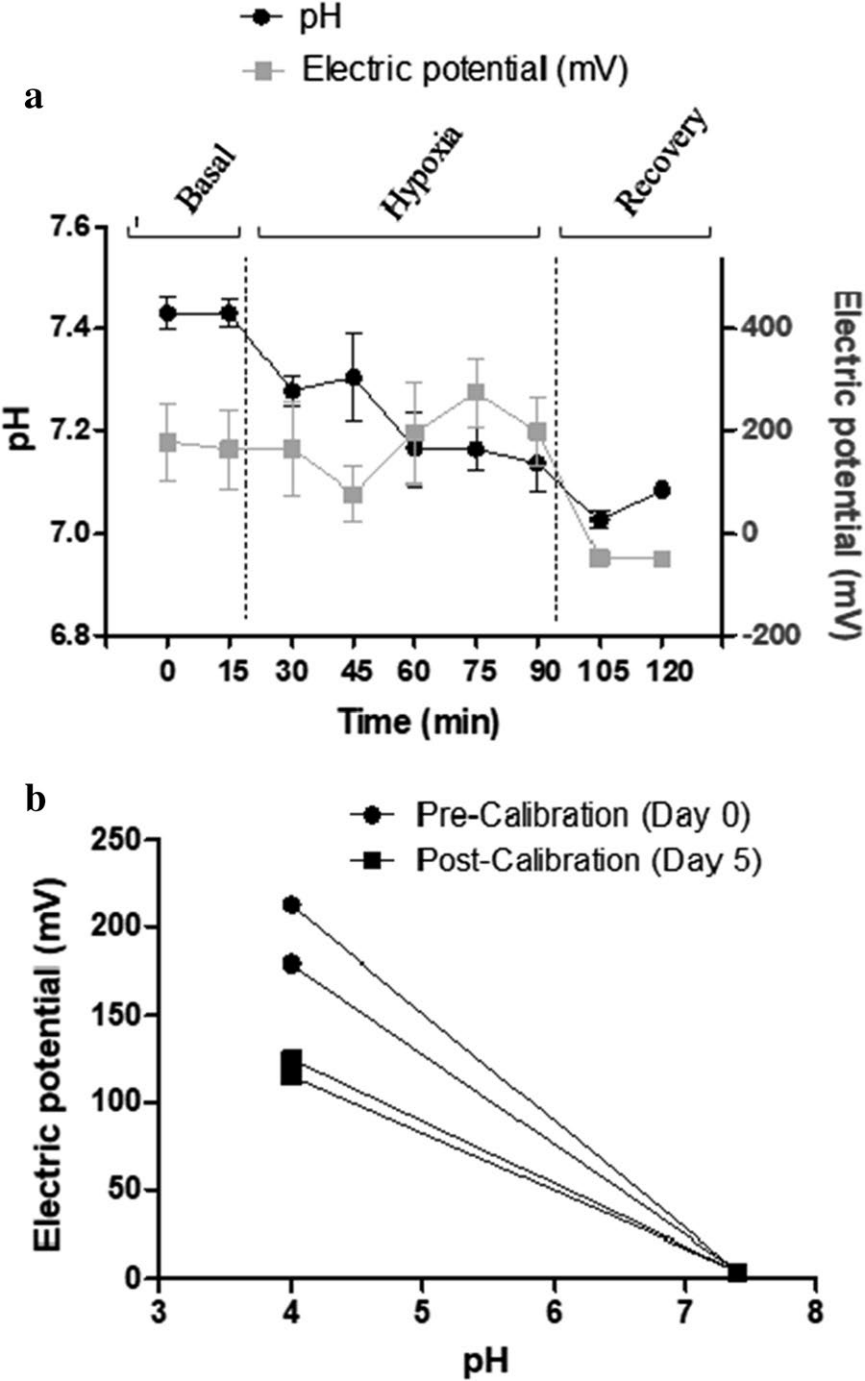
**a** Evolution of pH (measured by arterial gasometry) and electric potential (mV) (measured by pH electrochemical sensors) in the in vivo model during ventilatory hypoxia induction. **b** Pre- and post-calibration of the sensors in PBS buffer with pH values of 4.006 and 7.413. Data are expressed as mean ± SEM. *N* = 6 animals, 7 electrochemical sensors tested in vivo and 2 electrochemical sensors for post-calibration analyses

A description of the signal detected by the ex vivo and in vivo sensing during the different phases of the study is given in Fig. 5a. Overall, in the long-term evaluation, the gasometry analysis showed a similar pattern of pH changes to that reported in the short-term evaluation in the 3 phases of the ventilatory hypoxia induction protocol. The basal electric potential registered by the electrochemical sensor was different on day 0 (900–650 mV) and day 5 (600 to − 200 mV). During the hypoxia-acidosis period, coinciding with the pH decrease (between 15 and 90 min), the sensor signal increased 21% in the in vivo sensing, which is higher in comparison with the increase observed in the short-term evaluation (21 vs 5.6%, P < 0.001). Although this increase is more marked, the electric signal increased after 30 min of hypoxia-acidosis, whereas in the short-term evaluation the current signal increase was at the same moment as the pH changes. In the recovery phase, there was a significant decrease in the electrical signal, although the pH did not return to the basal values (Fig. 5a).

### Post-calibration of the electrochemical sensors in the long-term evaluation

The signal of the pH electrochemical sensors obtained in the post-calibration phase was significantly lower in comparison with the pre-calibration analyses when the sensor was immersed in standard commercial solutions at pH 4.006. However, the signal remained stable between pre-calibration and post-calibration when the sensors were immersed in standard commercial solutions at pH 7.413 (Fig. 5b).

All raw data obtained from the different experiments (acid-based metabolites, readings of the electrochemical sensors in the short- and long-term evaluations) are detailed in Additional file 1: Tables S1–S11.

### Histology

**Fig. 6:**
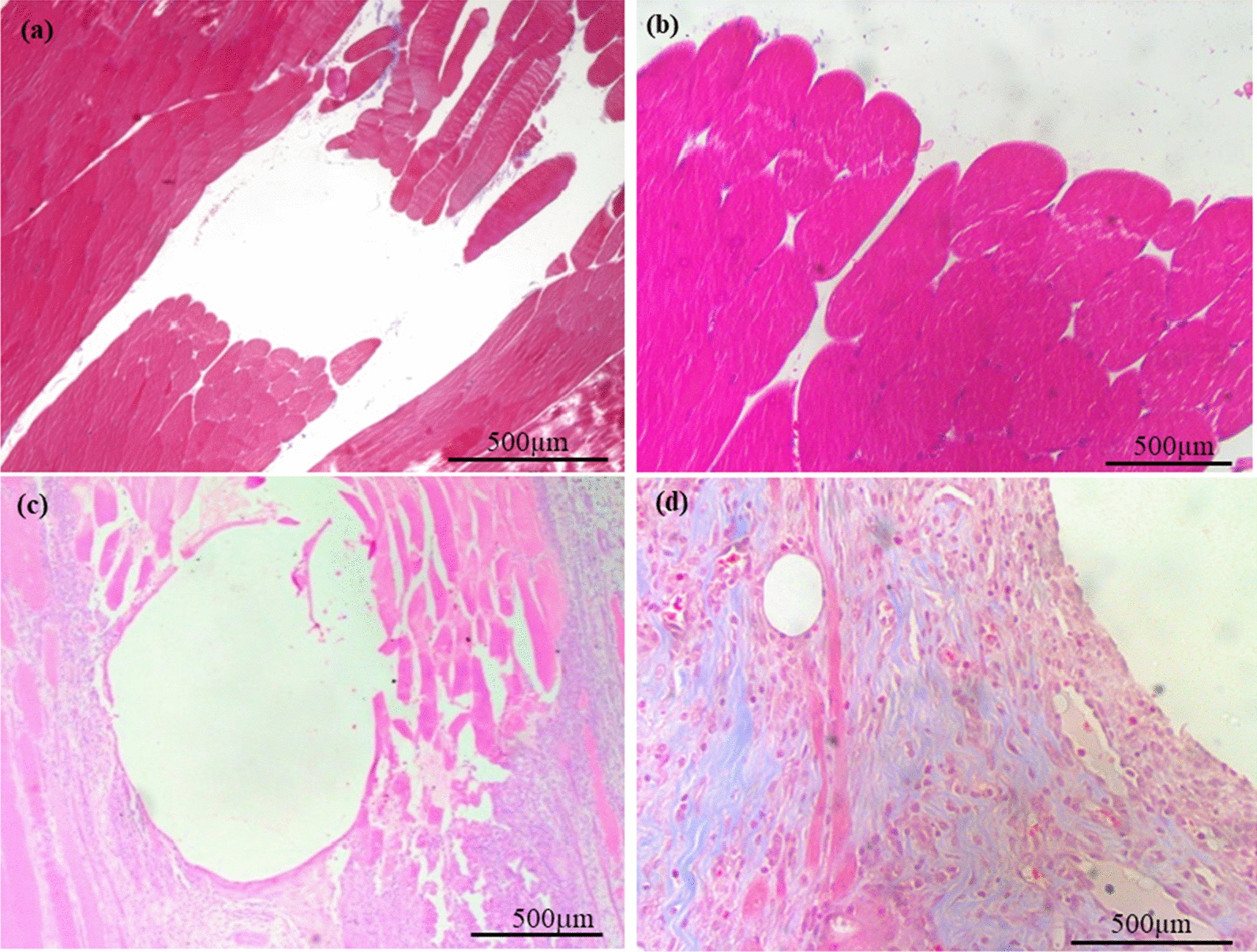
Representative images of hematoxylin/eosin (**a**, **c**) and Masson trichrome staining (**b**, **d**) of sensor implantation sites collected on the day of insertion (**a**, **b**) and 5 days after (**c**, **d**). **a** Normal muscular parenchyma delimitating the area of implantation without any inflammatory reaction at 5 ×; **b** Normal muscular parenchyma delimitating the area of implantation without any deposit of collagen fibers at × 20; **c** Muscular parenchyma delimitating the area of implantation with sub-acute inflammatory reaction at × 5; **d** Muscular parenchyma delimitating the area of implantation with deposit of collagen fibers at × 20. Inflammatory cell identification: Neutrophils: granulate cytoplasm; Monocytes: nucleus kidney shaped with foamy cytoplasm; Lymphocytes: dark nucleus with a small basophilic cytoplasm; giant multinucleated cells: large cells with multiple nucleus. Deposit of collagen observed as fibers stained with aniline blue

Samples collected on the day of insertion (day 0) showed no signs of alteration around the site of implantation. Normal muscular parenchyma without inflammatory reaction and with no deposition of collagen fibers was observed. In contrast, samples collected 5 days after insertion showed a sub-acute inflammatory reaction adjacent to the site of the implantation characterized by infiltration of neutrophils, monocytes, macrophage, lymphocytes, and multinucleated giant cells. Moreover, trichomic masson staining detected a mild deposition of collagen fibers (Fig. 6).

## Discussion

In this study, we report for the first time the performance of miniaturized electrochemical sensors to detect oxygen and pH changes early and to monitor these changes continuously in a model of ventilator hypoxia-acidosis. Overall, the electrochemical sensors used were able to detect changes in oxygen and pH when acute hypoxia-acidosis was induced on the same day of the electrochemical sensor insertion and especially in the blood sensing (ex vivo). In the long-term period, we observed a higher latency between gasometry changes and the electrochemical signal registered by the pH sensors.

### Ventilatory hypoxia induction protocol: Acid-based metabolites results

Different hypoxia-acidosis induction protocols in rabbits have been described, including either metabolic [26, 27] or respiratory [11, 28, 29] induction protocols. Following previous literature, hypoxia-acidosis was successfully achieved by combining a reduction of the fraction of inspired oxygen [14, 29], breaths per minute [26] and also the inhalatory flux.

The changes in acid–base metabolites observed in the ventilatory hypoxia induction protocol used were as expected. Oxygen was the metabolite for which the quickest and more marked changes were observed, followed by pH. Changes in the rest of the parameters (bicarbonate, potassium, and lactate) were more gradual and the values for these parameters did not return to the basal values in the recovery period. Changes in these last metabolites come from the cellular metabolism in response to the decrease in oxygen levels in the blood. This response includes several changes at cellular level making it a slower and more gradual response than that given to oxygen changes [30, 31]. The slower and gradual response could also be detected in the recovery period where after 30 min breathing of 100% oxygen air, these metabolites did not return to the basal values. Longer periods of recovery might be necessary to allow these metabolites to reach their basal values.

The selection of oxygen and pH metabolites for the electrochemical sensor monitoring was made considering the dynamicity of the changes reported in the gasometry analyses and also taking into consideration that both metabolites are considered to be hallmarks for the evaluation of hypoxic-acidotic status. Oxygen was the metabolite that most quickly reflected earlier changes in the acid–base status, whereas pH gives us information regarding the severity of hypoxia [32].

### Electrochemical sensors in the short-term evaluation

We have demonstrated that electrochemical sensors were able to detect oxygen and pH fluctuations, but with differences in their success rates. The oxygen sensor showed 100% success in the ex vivo sensing, although only half the electrochemical sensors inserted in the muscular tissue were able to detect signal. Similarly, 100% functional success was achieved in the pH ex vivo testing, with this percentage dropping to 70% in the short-term in vivo sensing. Success rate differences between ex vivo and in vivo sensing could be caused by the manipulation required for the in vivo insertion of the sensors. The insertion procedure could alter the functionality of the sensor surface with subsequent interference in the sensitivity of the sensors.

We also found differences in the basal signal of the electric current and electric potential between ex vivo and in vivo. The different behavior could be explained by differences in the diffusivity of oxygen and protons depending on whether the matrix surrounding the electrochemical sensor is blood or muscle tissue. It is known that blood and muscle present different electrolyte compositions [33] and depending on these, different electroactive species could also bind to the electrochemical sensor membrane, interfering with the final signal [23].

Regarding the electrochemical signal changes during the hypoxia-acidosis induction protocol, the performance is better in the ex vivo evaluations than in the in vivo evaluations. Electrochemical sensors detected the marked oxygen and pH decrease both ex vivo and in vivo, although the percentage of change in the sensor signal was higher when the sensor was immersed in blood (ex vivo) in comparison with the registered signal obtained from the sensors inserted in muscle (in vivo). In the recovery period, only the ex vivo electrochemical sensor was able to detect the changes in the oxygen and pH. Differences in the electrochemical signal obtained in the ex vivo and in vivo sensing could again be explained by differences in oxygen and pH diffusivity across tissues. Solid tissues such as muscle present a lower diffusivity of oxygen and pH in comparison with the vascular system, which is in constant flow and subject to constant renovation [34].

Finally, when we compared the performance of the oxygen and the pH electrochemical sensors, we observed higher detection by the oxygen sensor in both evaluations, in vivo and ex vivo. The fact that the pO_2_ levels can change more quickly and that these changes can be more pronounced than those in the pH levels could explain the better efficiency of the sensor in the detection of the oxygen fluctuations.

### Readings of the pH electrochemical sensor in the long-term evaluation

The electrochemical sensor performed differently in the short-term and long-term evaluations. First, the levels of the electric current in the basal period were different in the 2 evaluations. Secondly, in the hypoxia period, although the percentage of change in the registered electrochemical signal seemed to be higher in the long-term evaluation than in the short-term evaluation (20.99 vs 5.61%), the sensor signal in the long-term evaluation was delayed since it started to increase after 30 min of hypoxia. Finally, regarding the post-calibration, the electrochemical sensor transmitted a lower signal (mV) at the lower pH (4.006) after being inserted for 5 days. These differences in electric current, the delay in the electrochemical signal increase and the loss of sensibility in the post-calibration analyses could be due to changes in the tissue surrounding. Histological examination at day 0 demonstrated normal muscular parenchyma without any inflammation reaction or deposits of collagen fibers, whereas in the long-term period a sub-acute inflammatory reaction adjacent to the site of the implantation with inflammatory cells and deposit of collagen fibers was observed, similar to that previously observed for medical devices, prostheses and biomaterials [35]. The inflammation and collagen deposition may hamper proton diffusion through the permeable membrane and may thereby increase the latency of the electrochemical sensors in detecting the pH changes.

## Strengths and limitations

In this study we have demonstrated that miniaturized electrochemical sensors (diameter of ~ 500 µm) could detect oxygen and pH changes and monitor these changes continuously. Although different sensors have been already tested in vivo to detect hypoxia and acidosis [11, 20, 22], most of them are invasive or incompatible with being inserted in fetal tissue for long periods of time without compromising fetal wellbeing.

This study has also some limitations. First, the long-term functionality of the sensors needs to be improved. Experiments using the pH electrochemical sensors demonstrated several difficulties that should be overcome by reducing the inflammatory reaction associated with their implantation. The problems with the electrochemical signal after 5 days of implantation might be addressed by the use of more biocompatible material limiting the inflammatory reaction and collagen deposition. Secondly, the functionality of the electrochemical sensors in fetal tissue has not been evaluated in this study owing to the small size of rabbit fetuses. We do not expect changes in functionality related to implantation in fetal tissue; in any case, further studies with larger animal models (i.e., fetal lamb) are warranted. Finally, it should be considered that all comparisons in the in vivo evaluations have been performed between skeletal muscle (where the electrochemical sensors were implanted) and blood (using EPOC blood analysis), since there is not a gold standard that can be used to assess the acid-based metabolites in the skeletal muscle.

## Conclusions

In summary, this study provides experimental evidence that acute hypoxia-acidosis could be continuously monitored with miniaturized electrochemical sensors able to detect changes in oxygen and pH in the muscular tissue. Further research should focus on testing these miniaturized sensors in fetal tissue and should use different materials in order to improve oxygen and pH changes during longer periods of time. The continuous monitoring of fetal acid–base status would allow close surveillance of fetal wellbeing and also permit the establishment of clinical interventions aiming to decrease perinatal morbidity-mortality.

## Supplementary Information

**Additional file 1: Table S1.** Acid-based metabolites results during the ventilatory hypoxia induction protocol; **Table S2.** Electric current measured by oxygen electrochemical sensors during the ventilatory hypoxia induction protocol in the in vivo evaluation; **Table S3.** Electric current measured by oxygen electrochemical sensors during the ventilatory hypoxia induction protocol in the ex vivo evaluation; **Table S4.** Oxygen and pH results during the ventilatory hypoxia induction protocol from the animals used for ex vivo and in vivo oxygen electrochemical sensor evaluation; **Table S5.** Electric potential measured by pH electrochemical sensors during the ventilatory hypoxia induction protocol in the in vivo evaluation in the short-term period; **Table S6.** Electric potential measured by pH electrochemical sensors during the ventilatory hypoxia induction protocol in the ex vivo evaluation in the short-term period; **Table S7.** Oxygen and pH results during the ventilatory hypoxia induction protocol from the animals used for in vivo pH electrochemical sensor evaluation in the short-term period; **Table S8.** Oxygen and pH results during the ventilatory hypoxia induction protocol from the animals used for ex vivo pH electrochemical sensor evaluation in the short-term period; **Table S9.** Electric potential measured by pH electrochemical sensors during the ventilatory hypoxia induction protocol in the in vivo evaluation in the long-term period; **Table S10.** Oxygen and pH results during the ventilatory hypoxia induction protocol for the animals used for in vivo pH electrochemical sensor evaluation in the long-term period; **Table S11.** Pre and post-calibration of the pH electrochemical sensors inserted for the long-term evaluation.

## Data Availability

All data generated or analyzed during this study are included in this published article (and its supplementary information files).

## Abbreviations

pO_2_: Partial pressure of oxygen
$${\text{HCO3}}^{ - }$$: Bicarbonate
K +: Potassium
nA: Electric current
mV: Electric potential

## References

[1] Hutter D, Kingdom J, Jaeggi E (2010). Causes and mechanisms of intrauterine hypoxia and its impact on the fetal cardiovascular system: a review. Int J Pediatr.

[2] Lackman F, Capewell V, Gagnon R, Richardson B (2001). Fetal umbilical cord oxygen values and birth to placental weight ratio in relation to size at birth. Am J Obstet Gynecol.

[3] Nicolaides KH, Economides DL, Soothill PW (1989). Blood gases, pH, and lactate in appropriate- and small-for-gestational-age fetuses. Am J Obstet Gynecol.

[4] Soothill PW, Nicolaides KH, Campbell S (1987). Prenatal asphyxia, hyperlacticaemia, hypoglycaemia, and erythroblastosis in growth retarded fetuses. Br Med J (Clin Res Ed).

[5] Vyas S, Nicolaides KH, Bower S, Campbell S (1990). Middle cerebral artery flow velocity waveforms in fetal hypoxaemia. Br J Obstet Gynaecol.

[6] Baschat AA, Gembruch U (2002). Evaluation of the fetal coronary circulation. Ultrasound Obstet Gynecol.

[7] Baschat AA (2005). Arterial and venous Doppler in the diagnosis and management of early onset fetal growth restriction. Early Hum Dev.

[8] Bilardo CM, Nicolaides KH, Campbell S (1990). Doppler measurements of fetal and uteroplacental circulations: relationship with umbilical venous blood gases measured at cordocentesis. Am J Obstet Gynecol.

[9] Figueras F, Gratacós E (2014). Update on the diagnosis and classification of fetal growth restriction and proposal of a stage-based management protocol. Fetal Diagn Ther.

[10] Morgan C, Newell SJ, Ducker DA, Hodgkinson J, White DK, Morley CJ, Church JM (1999). Continuous neonatal blood gas monitoring using a multiparameter intra-arterial sensor. Arch Dis Child Fetal Neonatal Ed.

[11] Devlieger R, Gratacós E, Wu J, Yesildaglar N, Ghysel C, Barki G, Deprest J (2000). Continuous monitoring of fetal pH, pO2 and pCO2 using a fiberoptic multiparameter sensor in animal models reproducing in utero conditions. Fetal Diagn Ther.

[12] Formenti F, Farmery AD (2017). Intravascular oxygen sensors with novel applications for bedside respiratory monitoring. Anaesthesia.

[13] Jin W, Wu L, Song Y, Jiang J, Zhu X, Yang D (2011). Continuous intra-arterial blood pH monitoring by a fiber-optic fluorosensor. IEEE Trans Biomed Eng IEEE.

[14] Khan N, Hou H, Eskey CJ, Moodie K, Gohain S, Du G (2015). Deep-tissue oxygen monitoring in the brain of rabbits for stroke research. Stroke.

[15] Tsytsarev V, Akkenti F, Pumbo E, Tang Q, Chen Y, Erzurumlu RS (2017). Planar implantable sensor for in vivo measurement of cellular oxygen metabolism in brain tissue. J Neurosci Methods.

[16] Zhao F, Zhang L, Zhu A, Shi G, Tian Y (2016). In vivo monitoring of local pH values in a live rat brain based on the design of a specific electroactive molecule for H+. Chem Commun..

[17] Zhou J, Zhang L, Tian Y (2016). Micro electrochemical pH sensor applicable for real-time ratiometric monitoring of pH values in rat brains. Anal Chem.

[18] Weinstein DH, deRijke S, Chow CC, Foruraghi L, Zhao X, Wright EC (2013). A new method for determining gastric acid output using a wireless pH-sensing capsule. Aliment Pharmacol Ther..

[19] Wolpert PW, Noller K, Shaughnessy D, Houchin DN, Baccari ME, Miller FA (1970). Tissue pH: a new clinical tool. Arch Surg.

[20] Herdman KM, Breslin CB, Finnerty NJ (2019). Physiological monitoring of tissue pH: in vitro characterisation and in vivo validation of a quinone-modified carbon paste electrode. Electrochim Acta.

[21] Gottrup F, Firmin R, Rabkin J, Halliday BJ, Hunt TK (1987). Directly measured tissue oxygen tension and arterial oxygen tension assess tissue perfusion. Crit Care Med.

[22] Theodor M, Ruh D, Subramanian S, Forster K, Heilmann C, Beyersdorf F (2014). Implantable pulse oximetry on subcutaneous tissue. 36th Annu Int Conf IEEE Eng Med Biol Soc..

[23] Rivas L, Dulay S, Miserere S, Pla L, Marin SB, Parra J (2020). Micro-needle implantable electrochemical oxygen sensor: ex-vivo and in-vivo studies. Biosens Bioelectron.

[24] Ambrózy A, Hlavatá L, Labuda J (2013). Protective membranes at electrochemical biosensors. Acta Chim Slov.

[25] Clark LC, Wolf R, Granger D, Taylor Z (1953). Continuous recording of blood oxygen tensions by polarography. J Appl Physiol.

[26] Jin W, Jiang J, Wang X, Zhu X, Wang G, Song Y (2011). Continuous intra-arterial blood pH monitoring in rabbits with acid-base disorders. Respir Physiol Neurobiol.

[27] Khazaei M, Nematbakhsh M (2012). Experimentally-induced metabolic acidosis does not alter aortic fatty streak formation in high-cholesterol fed rabbits. Iran J Basic Med Sci.

[28] Baber SR, Li H, Simakajornboon N, Kadowitz PJ, Ross-Ascuitto NT, Ascuitto RJ (2005). Analysis of pulmonary vascular response to acute alveolar hypoxic challenge in young rabbits subjected to chronic hypoxia from birth. J Cardiovasc Pharmacol.

[29] Ward WK, Wood MD, Slobodzian EP (2002). Continuous amperometric monitoring of subcutaneous oxygen in rabbit by telemetry. J Med Eng Technol.

[30] Michiels C (2004). Physiological and pathological responses to hypoxia. Am J Pathol.

[31] Wheaton WW, Chandel NS (2011). Hypoxia regulates cellular metabolism. Am J Physiol Cell Physiol.

[32] Omo-Aghoja L (2014). Maternal and fetal acid-base chemistry: a major determinant of perinatal outcome. Ann Med Health Sci Res.

[33] Leighton HL (1990). Body composition, normal electrolyte concentrations, and the maintenance of normal volume, tonicity, and acid-base metabolism. Pediatr Clin North Am.

[34] Klaus S, Heringlake M, Gliemroth J, Pagel H, Staubach K, Bahlmann L (2003). Biochemical tissue monitoring during hypoxia and reoxygenation. Resuscitation.

[35] Anderson JM, Rodriguez A, Chang DT (2008). Foreign body reaction to biomaterials. Semin Immunol.

