# Supplementary material for: Non-invasive monitoring of pH and oxygen using miniaturized electrochemical sensors in an animal model of acute hypoxia
Source: J Transl Med. 2021 Feb 4;19:53. doi: 10.1186/s12967-021-02715-7 (PMC7863274; doi:10.1186/s12967-021-02715-7)
Supplement: Supplementary file 1 — Additional file 1: Table S1. Acid-based metabolites results during the ventilatory hypoxia induction protocol; Table S2. Electric current measured by oxygen electrochemical sensors during the ventilatory hypoxia induction protocol in the in vivo evaluation; Table S3. Electric current measured by oxygen electrochemical sensors during the ventilatory hypoxia induction protocol in the ex vivo evaluation; Table S4. Oxygen and pH results during the ventilatory hypoxia induction protocol from the animals used for ex vivo and in vivo oxygen electrochemical sensor evaluation; Table S5. Electric potential measured by pH electrochemical sensors during the ventilatory hypoxia induction protocol in the in vivo evaluation in the short-term period; Table S6. Electric potential measured by pH electrochemical sensors during the ventilatory hypoxia induction protocol in the ex vivo evaluation in the short-term period; Table S7. Oxygen and pH results during the ventilatory hypoxia induction protocol from the animals used for in vivo pH electrochemical sensor evaluation in the short-term period; Table S8. Oxygen and pH results during the ventilatory hypoxia induction protocol from the animals used for ex vivo pH electrochemical sensor evaluation in the short-term period; Table S9. Electric potential measured by pH electrochemical sensors during the ventilatory hypoxia induction protocol in the in vivo evaluation in the long-term period; Table S10. Oxygen and pH results during the ventilatory hypoxia induction protocol for the animals used for in vivo pH electrochemical sensor evaluation in the long-term period; Table S11. Pre and post-calibration of the pH electrochemical sensors inserted for the long-term evaluation. [file 12967_2021_2715_MOESM1_ESM.docx]

| **Time** | | **0** | **15** | **30** | **45** | **60** | **75** | **90** | **105** | **120** |
| --- | --- | --- | --- | --- | --- | --- | --- | --- | --- | --- |
|  |  | **Basal** | | **Hypoxia** | | | | | **Recovery** | |
| pO2  (mmHg) | nº values | 21 | 21 | 17 | 21 | 19 | 21 | 16 | 21 | 19 |
|  | Mean | 258.5 | 266.2 | 23.05 | 23.63 | 24.39 | 27.41 | 22.46 | 208.5 | 289.2 |
|  | SEM | 27.69 | 24.15 | 1.77 | 1.41 | 2.51 | 2.46 | 1.84 | 30.35 | 33.85 |
| pH | nº values | 24 | 25 | 16 | 22 | 20 | 21 | 19 | 18 | 19 |
|  | Mean | 7.45 | 7.45 | 7.33 | 7.26 | 7.15 | 7.14 | 7.12 | 7.10 | 7.18 |
|  | SEM | 0.02 | 0.01 | 0.02 | 0.03 | 0.04 | 0.03 | 0.03 | 0.04 | 0.03 |
| Lactate  (mmol/L) | nº values | 25 | 26 | 17 | 23 | 21 | 21 | 19 | 18 | 19 |
|  | Mean | 3.55 | 4.03 | 8.27 | 9.50 | 12.40 | 11.30 | 11.75 | 15.20 | 13.19 |
|  | SEM | 0.38 | 0.33 | 0.66 | 0.76 | 0.98 | 0.76 | 0.84 | 1.02 | 1.05 |
| HCO3-  (mmol/L) | nº values | 26 | 26 | 17 | 23 | 20 | 19 | 17 | 20 | 20 |
|  | Mean | 37.21 | 38.25 | 35.47 | 30.98 | 28.25 | 26.37 | 23.95 | 21.49 | 22.44 |
|  | SEM | 0.78 | 0.83 | 1.40 | 1.53 | 1.86 | 1.55 | 1.60 | 1.64 | 1.87 |
| K+  (mmol/L) | nº values | 26 | 25 | 17 | 23 | 20 | 20 | 17 | 19 | 20 |
|  | Mean | 4.67 | 4.81 | 5.56 | 5.68 | 5.79 | 5.87 | 5.82 | 6.19 | 6.89 |
|  | SEM | 0.17 | 0.19 | 0.25 | 0.18 | 0.23 | 0.23 | 0.21 | 0.39 | 0.42 |

# **Additional file 1**

**Table S1. Acid-based metabolites results during the ventilatory hypoxia induction protocol**

**Table S2. Electric current measured by oxygen electrochemical sensors during the ventilatory hypoxia induction protocol in the in vivo evaluation**

| **Time** | | **0** | **15** | **30** | **45** | **60** | **75** | **90** | **105** | **120** |
| --- | --- | --- | --- | --- | --- | --- | --- | --- | --- | --- |
|  |  | **Basal** | | **Hypoxia** | | | | | **Recovery** | |
| Electric current  (nA) | nº values | 4 | 3 | 4 | 6 | 3 | 2 | 2 | 5 | 4 |
|  | Mean | -923.3 | -1058.0 | -739.5 | -717.6 | -774.2 | -671.7 | -779.9 | -762.0 | -784.0 |
|  | SEM | 128.5 | 259.4 | 64.28 | 27.98 | 9.93 | 69.33 | 7.06 | 41.11 | 34.64 |

**Table S3. Electric current measured by oxygen electrochemical sensors during the ventilatory hypoxia induction protocol in the ex vivo evaluation**

| **Time** | | **0** | **15** | **30** | **45** | **60** | **75** | **90** | **105** | **120** |
| --- | --- | --- | --- | --- | --- | --- | --- | --- | --- | --- |
|  |  | **Basal** | | **Hypoxia** | | | | | **Recovery** | |
| Electric current  (nA) | nº values | 3 | 3 | 4 | 5 | 4 | 2 | 1 | 5 | 5 |
|  | Mean | -227.9 | -243.8 | -39.05 | -19.47 | 11.95 | -43.41 | -13.71 | -227.3 | -256.9 |
|  | SEM | 132.4 | 88.16 | 10.22 | 1.631 | 27.03 | 36.35 | 0.0 | 79.21 | 86.17 |

**Table S4. Oxygen and pH results during the ventilatory hypoxia induction protocol from the animals used for ex vivo an in vivo oxygen electrochemical sensor evaluation**

| **Time** | | **0** | **15** | **30** | **45** | **60** | **75** | **90** | **105** | **120** |
| --- | --- | --- | --- | --- | --- | --- | --- | --- | --- | --- |
|  |  | **Basal** | | **Hypoxia** | | | | | **Recovery** | |
| pO2  (mmHg) | nº values | 3 | 3 | 4 | 5 | 4 | 4 | 1 | 5 | 6 |
|  | Mean | 383.5 | 374.1 | 31.08 | 26.44 | 21.13 | 25.50 | 32.20 | 369.4 | 418.5 |
|  | SEM | 118.5 | 70.59 | 4.948 | 4.578 | 4.480 | 4.859 | 0.0 | 54.26 | 34.60 |
| pH | nº values | 3 | 3 | 4 | 5 | 4 | 4 | 1 | 5 | 6 |
|  | Mean | 7.56 | 7.52 | 7.37 | 7.31 | 7.14 | 7.20 | 7.28 | 7.25 | 7.28 |
|  | SEM | 0.02 | 0.03 | 0.04 | 0.04 | 0.04 | 0.05 | 0.0 | 0.07 | 0.04 |

Table S5. Electric potential measured by pH electrochemical sensors during the ventilatory hypoxia induction protocol in the in vivo evaluation at short term period

| **Time** | | **0** | **15** | **30** | **45** | **60** | **75** | **90** | **105** | **120** |
| --- | --- | --- | --- | --- | --- | --- | --- | --- | --- | --- |
|  |  | **Basal** | | **Hypoxia** | | | | | **Recovery** | |
| Electric potential  (mV) | nº values | 7 | 6 | 5 | 5 | 4 | 3 | 3 | 2 | 1 |
|  | Mean | 786.6 | 797.3 | 880.1 | 841.5 | 863.7 | 857.0 | 842.0 | 871.5 | 768.5 |
|  | SEM | 25.41 | 24.77 | 6.660 | 31.77 | 24.12 | 40.30 | 35.67 | 28.53 | 0.0 |

**Table S6. Electric potential measured by pH electrochemical sensors during the ventilatory hypoxia induction protocol in the ex vivo evaluation at short term period**

| **Time** | | **0** | **15** | **30** | **45** | **60** | **75** | **90** | **105** | **120** |
| --- | --- | --- | --- | --- | --- | --- | --- | --- | --- | --- |
|  |  | **Basal** | | **Hypoxia** | | | | | **Recovery** | |
| Electric potential  (mV) | nº values | 3 | 2 | 1 | 1 | 2 | 3 | 3 | 1 | 1 |
|  | Mean | 48.77 | 39.47 | 50.66 | 63.38 | 55.47 | 59.33 | 55.00 | 70.94 | 56.00 |
|  | SEM | 6.115 | 14.53 | 0.0 | 0.0 | 13.53 | 14.37 | 14.35 | 0.0 | 0.0 |

| **Time** | | **0** | **15** | **30** | **45** | **60** | **75** | **90** | **105** | **120** |
| --- | --- | --- | --- | --- | --- | --- | --- | --- | --- | --- |
|  |  | **Basal** | | **Hypoxia** | | | | | **Recovery** | |
| pO2  (mmHg) | nº values | 9 | 9 | 8 | 5 | 6 | 3 | 3 | 5 | 4 |
|  | Mean | 276.1 | 334.1 | 23.45 | 31.26 | 30.42 | 17.63 | 16.5 | 195.0 | 206.2 |
|  | SEM | 42.82 | 42.16 | 2.355 | 8.068 | 7.149 | 4.454 | 3.995 | 72.68 | 99.63 |
| pH | nº values | 9 | 9 | 8 | 7 | 6 | 4 | 5 | 2 | 2 |
|  | Mean | 7.45 | 7.45 | 7.27 | 7.21 | 7.18 | 7.12 | 7.14 | 7.15 | 7.12 |
|  | SEM | 0.03 | 0.03 | 0.07 | 0.09 | 0.05 | 0.09 | 0.06 | 0.17 | 0.05 |

**Table S7. Oxygen and pH results during the ventilatory hypoxia induction protocol from the animals used for in vivo pH electrochemical sensor evaluation at short term period**

**Table S8. Oxygen and pH results during the ventilatory hypoxia induction protocol from the animals used for ex vivo pH electrochemical sensor evaluation at short term period**

| **Time** | | **0** | **15** | | **30** | **45** | **60** | **75** | **90** | **105** | **120** |
| --- | --- | --- | --- | --- | --- | --- | --- | --- | --- | --- | --- |
|  |  | **Basal** | | | **Hypoxia** | | | | | **Recovery** | |
| pO2  (mmHg) | nº values | 3 | | 3 | 2 | 2 | 3 | 2 | 2 | 2 | 1 |
|  | Mean | 276.50 | | 245.20 | 25.75 | 41.0 | 23.73 | 18.40 | 12.65 | 169.40 | 122.40 |
|  | SEM | 22.51 | | 36.84 | 2.250 | 20.90 | 3.367 | 7.600 | 1.850 | 55.00 | 0.0 |
| pH | nº values | 3 | | 3 | 2 | 2 | 3 | 3 | 2 | 1 | 1 |
|  | Mean | 7.43 | | 7.39 | 7.22 | 7.26 | 7.16 | 7.16 | 7.20 | 6.98 | 7.07 |
|  | SEM | 0.03 | | 0.03 | 0.05 | 0.10 | 0.09 | 0.12 | 0.15 | 0.0 | 0.0 |

Table S9. Electric potential measured by pH electrochemical sensors during the ventilatory hypoxia induction protocol in the in vivo evaluation at long term period

| **Time** | | **0** | **15** | **30** | **45** | **60** | **75** | **90** | **105** | **120** |
| --- | --- | --- | --- | --- | --- | --- | --- | --- | --- | --- |
|  |  | **Basal** | | **Hypoxia** | | | | | **Recovery** | |
| Electric potential  (mV) | nº values | 7 | 7 | 6 | 2 | 5 | 7 | 7 | 2 | 2 |
|  | Mean | 177.1 | 162.9 | 164.3 | 76.88 | 195.1 | 274.2 | 197.1 | -46.93 | -49.69 |
|  | SEM | 75.21 | 75.58 | 89.78 | 52.60 | 100.9 | 67.47 | 68.84 | 10.27 | 9.280 |

**Table S10. Oxygen and pH results during the ventilatory hypoxia induction protocol from the animals used for in vivo pH electrochemical sensor evaluation at long term period**

| **Time** | | **0** | **15** | **30** | **45** | **60** | **75** | **90** | **105** | **120** |
| --- | --- | --- | --- | --- | --- | --- | --- | --- | --- | --- |
|  |  | **Basal** | | **Hypoxia** | | | | | **Recovery** | |
| pO2  (mmHg) | nº values | 5 | 6 | 4 | 3 | 4 | 6 | 6 | 2 | 2 |
|  | Mean | 193.7 | 232.6 | 22.98 | 20.47 | 21.05 | 22.64 | 21.95 | 110.1 | 153.2 |
|  | SEM | 47.03 | 39.43 | 1.39 | 0.72 | 1.86 | 2.19 | 0.52 | 15.40 | 55.75 |
| pH | nº values | 5 | 6 | 4 | 3 | 4 | 7 | 6 | 2 | 2 |
|  | Mean | 7.43 | 7.43 | 7.28 | 7.30 | 7.17 | 7.16 | 7.14 | 7.03 | 7.09 |
|  | SEM | 0.03 | 0.03 | 0.03 | 0.09 | 0.07 | 0.04 | 0.05 | 0.02 | 0.01 |

| **pH** | | **4** | | **7.4** | |
| --- | --- | --- | --- | --- | --- |
|  |  | **Pre-calibration** | **Post-calibration** | **Pre-calibration** | **Post-calibration** |
| Electric potential  (mV) | nº values | 2 | 2 | 2 | 2 |
|  | Mean | 195.9 | 120.1 | 3.600 | 3.600 |
|  | SEM | 16.67 | 5.045 | 0.0 | 0.0 |

**Table S11. Pre and post-calibration of the pH electrochemical sensors inserted for long term evaluation**
